# Scanning transmission imaging in the helium ion microscope using a microchannel plate with a delay line detector

**DOI:** 10.3762/bjnano.11.167

**Published:** 2020-12-11

**Authors:** Eduardo Serralta, Nico Klingner, Olivier De Castro, Michael Mousley, Santhana Eswara, Serge Duarte Pinto, Tom Wirtz, Gregor Hlawacek

**Affiliations:** 1 Institute of Ion Beam Physics and Materials Research, Helmholtz-Zentrum Dresden-Rossendorf, Bautzner Landstr. 400, 01328 Dresden, Germany; 2 Technische Universität Dresden, Dresden 01069, Germany; 3 Advanced Instrumentation for Nano-Analytics (AINA), MRT Department, Luxembourg Institute of Science and Technology, 41, rue du Brill, L-4422, Belvaux, Luxembourg; 4 Photonis Netherlands B.V., Dwazziewegen 2, 9301 ZR Roden, Netherlands

**Keywords:** bright-field, channeling, dark-field, delay line detector, helium ion microscopy, scanning transmission ion microscopy

## Abstract

A detection system based on a microchannel plate with a delay line readout structure has been developed to perform scanning transmission ion microscopy (STIM) in the helium ion microscope (HIM). This system is an improvement over other existing approaches since it combines the information of the scanning beam position on the sample with the position (scattering angle) and time of the transmission events. Various imaging modes, such as bright field and dark field or the direct image of the transmitted signal, can be created by post-processing the collected STIM data. Furthermore, the detector has high spatial and temporal resolution, is sensitive to both ions and neutral particles over a wide energy range, and shows robustness against ion beam-induced damage. A special in-vacuum movable support gives the possibility of moving the detector vertically, placing the detector closer to the sample for the detection of high-angle scattering events, or moving it down to increase the angular resolution and distance for time-of-flight measurements. With this new system, we show composition-dependent contrast for amorphous materials and the contrast difference between small-angle and high-angle scattering signals. We also detect channeling-related contrast on polycrystalline silicon, thallium chloride nanocrystals, and single-crystalline silicon by comparing the signal transmitted at different directions for the same data set.

## Introduction

The helium ion microscope (HIM) is an instrument that has already proven its value for high-resolution imaging, compositional analysis, nanofabrication, and materials modification [1–2]. It generates a focused helium (or neon) ion beam with sub-nanometer spot size and rasters it across the sample. The beam can be used for both imaging and modification of samples at the nanometer scale. The standard and most widely used imaging mode in the HIM is using an Everhart–Thornley detector (ET) [3] for collecting secondary electrons (SEs) emitted from the top surface of the sample, which carry mainly topographic information [4].

Other detectors and signals have been used to expand the capabilities of the HIM. Imaging with back-scattered particles [5–6] can add compositional information and reveal buried structures [7]. Ionoluminescence has been studied by detecting the light emitted from the sample during ion bombardment [8–10]. Moreover, compositional analyses using secondary ion mass spectrometry (SIMS) can be performed in the HIM with a lateral resolution of the order of 10 nm [11–14].

Transmission-mode imaging can further improve the capabilities of the HIM since it depends on different contrast mechanisms and gives information on sub-surface features as well. There are several ways of using the transmitted signal to form an image. In thin samples, the helium bean also generates SEs that can leave the sample from the bottom surface. This type of SEs was already used as an imaging signal [15]. More commonly, transmission imaging signals depend on the particles that pass through the sample and on how they are scattered. In bright-field (BF) mode, the image is produced by mapping the part of the beam that has suffered very little, or no, deflection. In dark-field (DF) mode, the deflected part of the beam is used to generate the signal for the image. In annular dark-field (ADF) mode, the beam transmitted at a particular polar angle region is integrated over a complete annulus. Alternatively, the image can be formed using the beam deflected in a polar and azimuthal angular sector.

For amorphous materials under perpendicular incidence, the transmitted beam is expected to be scattered symmetrically around the axis of incidence. The average polar angle of scattering depends on both the material and the thickness of the sample. Different materials and thickness combinations create distinct polar-angle distributions of scattering, producing a contrast similar to the mass-thickness contrast in transmission electron microscopy. In BF mode, the areas of the sample with little or no scattering appear with high intensity in the image, and regions of the sample that scatter more than the collection angle of the detector will appear with low intensity. In a complementary manner, in ADF mode, the areas of the sample that scatter to the considered angular interval will appear bright in the image, and the areas of the sample with little scattering will appear dark. BF imaging has the advantage of having higher count rates for the same beam current in thin samples. On the other hand, by adjusting the collection angles to fit the maximum of this distribution for a given material and thickness, ADF imaging can enhance the contrast of certain compositional features of the sample.

Crystalline materials can also give rise to additional contrast mechanisms. In crystalline materials, the stopping force depends on the orientation of the crystal [16]. Along some orientations, the target atoms are aligned in rows or planes, thereby creating easier directions for the penetration of the projectile atom. If the projectile atom reaches the crystal at an angle smaller than the critical angle for such an axial or planar channeling direction, the projectile will be steered along this direction and will experience a reduced probability of undergoing large-angle scattering. Hence, it will have a smaller energy loss per distance compared to random directions. This phenomenon is called the channeling effect and has been described for megaelectronvolt ions in detail in [17]. When compared to a random orientation, the channeling directions also have reduced secondary electron [18], back-scattering and sputter yields. Conversely, the ions have increased range and transmission probability in these directions. Channeling contrast in the HIM was demonstrated using SE imaging [18–19] and using the back-scattering signal [20]. The channeling effect in the HIM has also been studied using Monte Carlo [21] and molecular dynamics [22] simulations.

Measuring the energy of the transmitted particles is a novel technique that adds an information channel to the previously discussed transmission imaging modes. It will provide information on the phenomena occurring during the projectile–target interaction and can increase the signal-to-noise ratio [23]. Since most likely a considerable fraction of the transmitted particles at this energy range is neutral [24–25], magnetic or electrostatic spectrometers cannot be used. Therefore, ion energy-loss spectrometry and energy-resolved imaging require an energy-sensitive detector or a detection system in which time-of-flight (ToF) measurements can be implemented.

Likewise, the use of the transmission signal in the HIM for visualizing diffraction patterns is, in theory, possible but has not been reported yet. Diffraction patterns yield additional information on the crystal lattice and orientation. However, this application demands a detector with high spatial resolution taking into consideration the energy range and typical space restrictions in the HIM.

In the past, several attempts to utilize the transmission signal in the HIM have been made. One approach is converting the transmitted particles into SEs by positioning a material with high SE yield below the sample and using the ET detector to collect the SEs coming from this material. This method has been used in BF mode for assessing the thickness of milled materials in the microscope [26–27] and has also been implemented for ADF imaging [15,28]. Mass-thickness contrast and thickness-fringes contrast have been shown in transmission mode in the HIM using this approach with a combined bright- and dark-field conversion detector [29]. Another approach using an annular microchannel plate detector was used for investigating gold–silica core–shell nanoparticles in ADF mode [30]. These approaches require a physical aperture to restrict the acceptance angle when performed in BF, and a physical change of the distance between the sample and the annular detector to adjust the acceptance angle interval when performed in DF. Most recently, a position-sensitive detector consisting of a silicon diode array has also been adopted for use in the HIM [31]. Later the same group also studied channeling effects on single-crystalline silicon with this detector [32].

In this work, we present a new system for comprehensive scanning transmission ion microscopy (STIM) analyses that gives more flexibility to the user than the earlier approaches. We adopted a microchannel plate (MCP) and a delay line readout structure as a position-sensitive detector to be used in the HIM. A special in-vacuum detector support allows one to mechanically control the acceptance angle during analysis. The resulting system has high spatial resolution and can be positioned to detect polar angles of deflection from 0 to 19°, with an angular resolution always better than 0.0033°. The selection of the transmission imaging mode and further tuning of acceptance angles can be done in post-processing. Additionally, ToF-resolved recording of the transmission events can be integrated into this system. Here, we use this system to study the mass-thickness-dependent contrast in amorphous materials and demonstrate transmission-channeling contrast using polycrystalline silicon, thallium chloride samples and beam steering in single-crystalline silicon.

## Experimental

**Figure 1:**
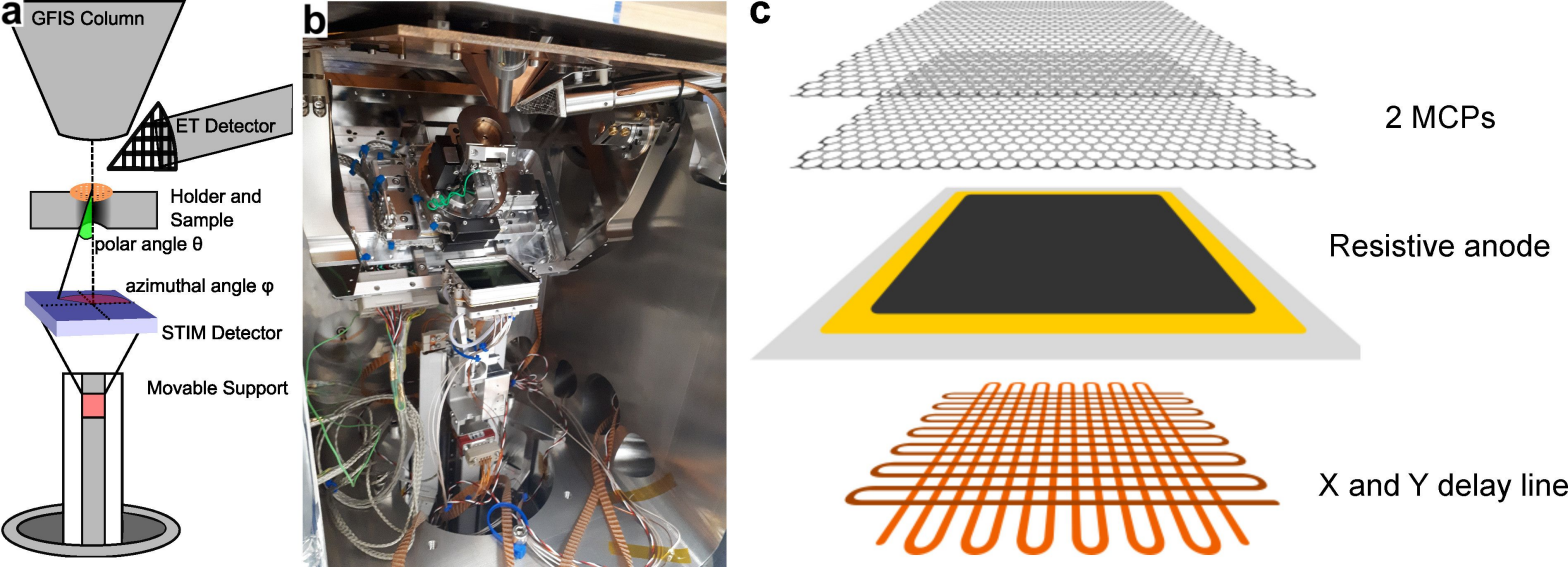
(a) Schematic representation of the STIM experiment. (b) Picture of the inner part of the chamber. (c) Schematic representation of the STIM detector.

The new STIM detector comprises a stack of two MCPs and a resistive anode layer with a delay line readout structure behind it, as represented in Figure 1c. The combination of MCPs with a delay line readout structure as a position-sensitive detector was first implemented for the detection of 1–15 keV electrons [33]. Since then, it has been used in many other applications such as in astrophysics [34], transmission electron microscopy [35], and hard X-ray photoelectron spectroscopy [36].

The working principle of the detector can be summarized as follows: At the front side of the MCP, SEs are produced by the impact of the impinging energetic particles (He ions and atoms). These SEs are drawn into the microchannels due to the applied bias. The number of SEs is multiplied by numerous collisions along the way within the channels creating an electron cloud. The electron cloud hits the resistive anode layer in front of the delay lines and, by capacitive coupling, induces signals on the delay line meanders. These signals are collected at the endpoints of each delay line and passed through separate constant-fraction discriminators. Finally, with a time-to-digital converter, the position of the cascade is computed by comparing the time of arrival of the pulses at the ends of each delay line. This can be performed with picosecond accuracy and thus makes the detector ideally suited for future ToF applications.

In the present form of the detector, we use two MCPs, each with a 50 mm by 50 mm square active area, stacked and rotated by 90° to each other with a gap of 100 μm between them. The first MCP has a magnesium oxide coating to increase the SE yield [37]. The MCP pores have a diameter of 25 μm and a center-to-center spacing of 32 μm. The bias angle of the pores is 16°. The combination of delay line readout performance and MCP characteristics results in a spatial resolution of 47.2 μm in the *x*-direction and 58.1 μm in the *y*-direction. Effectively, the detector has approximately 4 megapixels over its entire area. The MCP front is biased to a potential of approximately −2 kV, while the MCP back is kept at approximately −400 V, relative to the anode, which is at ground potential. With this bias scheme, in the working instrument (detectors and gauges powered up, column isolation valve closed) at a sample chamber pressure of 2 × 10^−7^ mbar, we measured a dark-count rate of 80 cps. The delay line readout has a specified maximum count rate of 5 Mcps for randomly distributed events.

However, in practice, the count rate is limited by the non-random nature of the transmitted particles. In thin light samples, most of the transmitted particles will hit the center of the detector. The pores of the MCP have an individual recharging time of the order of 0.5 ms, estimated considering the MCP pores as parallel capacitors and resistors. This results in the fact that a single channel can only correctly detect count rates lower than 2 kcps or currents smaller than 0.3 fA. In addition to that, given that the position on the detector is calculated based on the time difference between the signals, when dealing with multiple simultaneous events, there might be multiple solutions for the combinations of position and time. Therefore, when trying to compute events that are too close in time and position, the delay line structure algorithm might produce imaging artifacts with shapes reflecting these multiple solutions for multi-hit events.

For each detected event, the STIM data consists of the position on the detector, the position of the beam on the sample, and a time reference to an internal or external signal (used in ToF mode). The beam position on the sample is controlled by an external scan generator, which also provides the beam position to the detection hardware. The acquisition of the STIM data and the external scan generator are controlled by a LabView interface based on an earlier implementation used for ToF-SIMS in the HIM [14]. The program allows for the generation of sample images in which the gray value, or color, of each pixel is assigned based upon the strength of the signal striking the transmission detector or selected sub-regions thereof. In any case, we store a comprehensive data set containing the 2D location where the beam is rastered over the sample, together with the 2D location where the transmitted helium strikes the detector, and the ToF information. Therefore, the user can generate BF, ADF, or other DF images through post-processing the transmission data at will at any time.

The experiments were conducted using the npSCOPE prototype, which is a high-vacuum instrument based on the gas field ion source (GFIS) column technology. This instrument combines the helium ion microscopy techniques with SIMS using a magnetic sector spectrometer, STIM with this new detector, and cryo-microscopy capabilities in a single instrument and will be described in detail elsewhere. In comparison to a commercial HIM, this microscope has a larger vacuum chamber that allows for the installation of the STIM detector and its movable support. A schematic representation of the measuring geometry is displayed in Figure 1a together with an overview image of the STIM detector, the stage without the sidewall, the adapted sample holder, cryo-shields, and ion optical column (Figure 1b).

An in-vacuum movable linear support is used to control the detector distance to the sample. This means that the distance can be chosen to give the best compromise between maximum collection angle for high-angle scattering events, and angular resolution with longer distance (and time-of-flight) for higher energy resolution. The support consists of a vertically mounted movable rail, on which a carriage supporting the detector can travel up and down. The rail is mounted on a flange attached around the pumping hole of the chamber. The motion is driven by piezo motors (Nanomotion HR-8), and controlled by a motion controller (Nanomotion XCDX) using a closed feedback loop with optically encoded linear rails (Schneeberger Miniscale Plus). This construction is compatible with the high-vacuum requirements, is self-locking, requires no mechanical feedthroughs nor lubricants, and provides high accuracy regarding the position of the detector (down to 100 nm). In the npSCOPE prototype, the distance between the detector and the sample can be adjusted from 101 to 496 mm, with the closest position being limited by the current stage. In the highest vertical position, the detection of 13.9° polar deflection angles is possible for any azimuthal angle, or up to 19° in selected azimuthal angles corresponding to the corners of the square detector.

The sample is currently mounted in a way similar to the one presented in [31]. A sample holder with an extension arm with a hole is used to mount the sample. Since the extension arm is attached at 45°, the stage has to be tilted so that the sample can be aligned with the column axis. In order to allow the transmitted particles to reach the detector, we removed the sidewall of the cradle of the current stage. With a new dedicated stage design (currently under construction), the detector can reach a minimum distance to the sample of 50 mm, achieving maximum polar angles of 25° for any azimuthal angle, or up to 33° in the corners of the square detector. The detector support is designed in a way that it can be adapted and installed into the commercially available Orion NanoFab chamber, with a reduced travel range.

The images presented in this work in transmission mode, unless stated differently, were taken while operating the microscope at 30 kV acceleration voltage, with a 10 μm aperture, in spot control 6 (crossover position of −247 mm) and gun gas pressure of 5 × 10^−7^ mbar. These conditions provide an estimated beam current of 50 fA. For the STIM images, a single scan with pixel dwell time of 110 μs was used. In the SE imaging mode, we used the line average mode with ten scans and a pixel dwell time of 10 μs. The research data used in this publication is available in an open access repository [38].

## Results and Discussion

### Mass-thickness contrast

#### Bright-field and dark-field contrast

**Figure 2:**
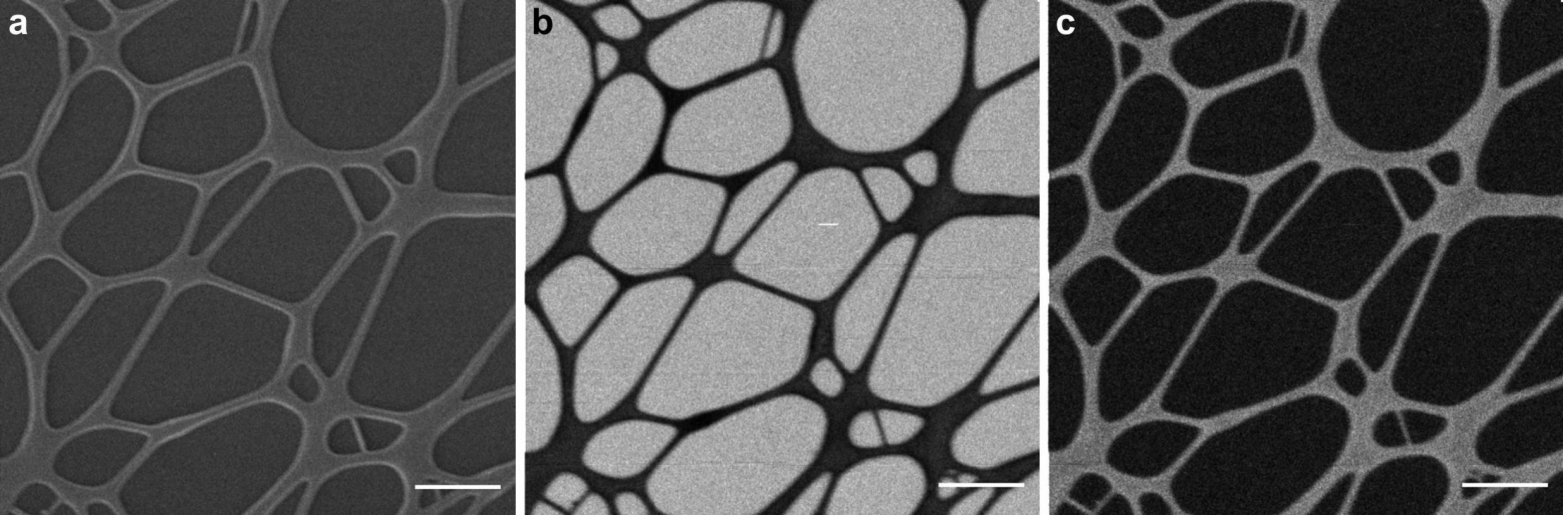
Micrographs of lacey carbon on carbon film. (a) Secondary electron imaging mode, (b) bright-field STIM image, with collection angle from 0 to 3°, and (c) annular dark-field STIM image, with collection angle from 8 to 13.9°. The scale bars are 1 μm.

In Figure 2, we show images of a carbon film under lacey carbon using the SE imaging mode (Figure 2a), BF STIM (Figure 2b), and ADF STIM (Figure 2c). The data for Figure 2b and Figure 2c was acquired in a single acquisition after Figure 2a, and the two images were generated selecting from the data two appropriate angular sectors of the detector.

The areas of the sample where the carbon film has the lacey carbon on top show different gray levels compared to the areas where there is only a homogeneous film. In general, since the average polar angle of scattering increases with the thickness, correctly adjusting the cut-off angle for the BF image can effectively suppress the signal from thicker areas of the sample in the final image. In contrast, in ADF thin areas of the sample are suppressed, while thicker regions appear bright if an appropriate minimum angle is chosen.

Here, we show two STIM images with different contrast using the same data set. During post-processing, the discrimination between ADF and BF has been done by choosing different minimum and maximum scattering angles for each image in order to maximize the contrast for each of them individually. Scattering angles between 0° and 3° have been used for BF while only scattered particles with scattering angles between 8 and 13.9° have been used for the corresponding ADF. The contrast due to the difference in thickness of the material can be noticed in these images.

#### Quantitative analysis

**Figure 3:**
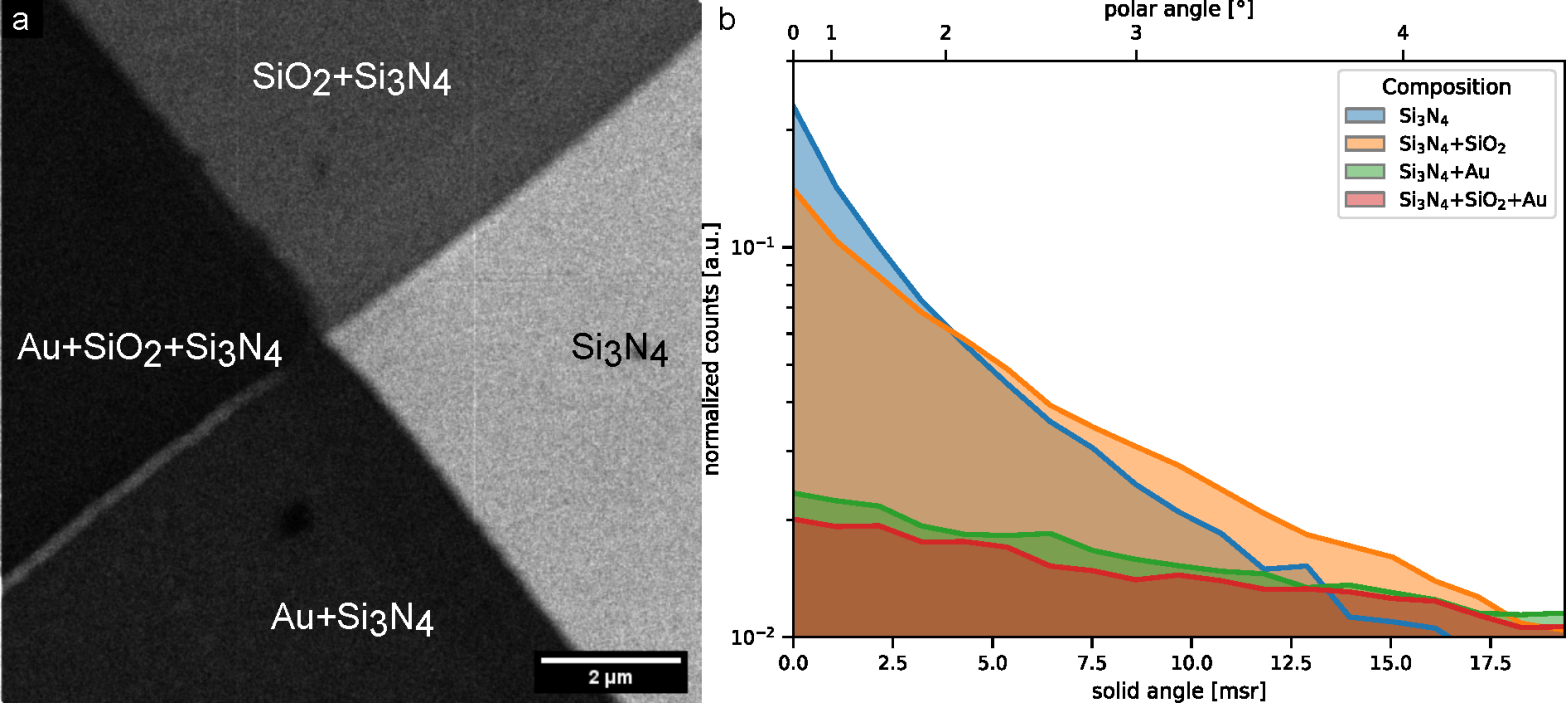
Bright-field image showing contrast due to the dependence of the exit angle on the material and the thickness of the layer. (a) Bright-field STIM image with collection angle from 0 to 4.5° of a silicon nitride membrane with silicon dioxide deposited on the upper left half and gold deposited on the lower left half. (b) TRIDYN simulation of the angular distribution of the transmitted beam.

Figure 3a is a bright-field image of a multilayer sample used to study STIM contrast using combinations of light and heavy elements. In the BF image (Figure 3a BF angles: 0 to 4.5°), we can clearly differentiate all four regions based on their gray levels. For this image we used 30 kV acceleration voltage, with a 5 μm aperture, in spot control 5, a gun gas pressure of 1.3 × 10^−6^ mbar, and 300 μs pixel dwell time.

The sample comprises a 20 nm thick silicon nitride membrane used as a support layer. A 20 nm thick layer of silicon dioxide was deposited on one half, visible on the upper left half of the area in Figure 3a. Then, in the next step, a gold layer of 20 nm was deposited on the lower left half of the sample, creating four distinct areas on the window. The different material stacks are indicated in the STIM image. In Figure 3b, we show simulations of the exit angular distribution of 30 keV He for the different stacks of materials that are present in the sample, using TRIDYN [39] in static mode. The graph presented in Figure 3b shows the corresponding transmission angular distribution for the region used in Figure 3a. The expected contrast between different areas of the sample for the detection range of 0 to 4.5° is calculated from these distributions. In Table 1, a comparison between the contrast calculated in the simulations and the contrast obtained from Figure 3a is given.

**Table 1:** Bright-field STIM contrast comparison: Intensity of the transmitted signal from 0 to 4.5°.

Material	Si_3_N_4_	SiO_2_ + Si_3_N_4_	Au + Si_3_N_4_	Au + SiO_2_ + Si_3_N_4_

Average counts per pixel	45.73	19.03	7.42	4.78
Experimental signal normalized	1	0.42	0.16	0.10
Simulated signal normalized	1	0.67	0.12	0.11

For this sample, the simulated and experimental contrast match qualitatively. A quantitative analysis shows relevant differences in the intensity levels of the regions. The relative intensity level of the area with the layer of silicon dioxide on top of the silicon nitride differs considerably in experiment and simulation. The signal in the area on which only gold is deposited is stronger than expected while the signal on the area on which only silicon dioxide is deposited is weaker. A further study on the thickness of each layer using different techniques has not been performed, although deviations of the layer thickness could be responsible for the observed mismatch.

### Beam steering and channeling

#### Polycrystalline silicon

**Figure 4:**
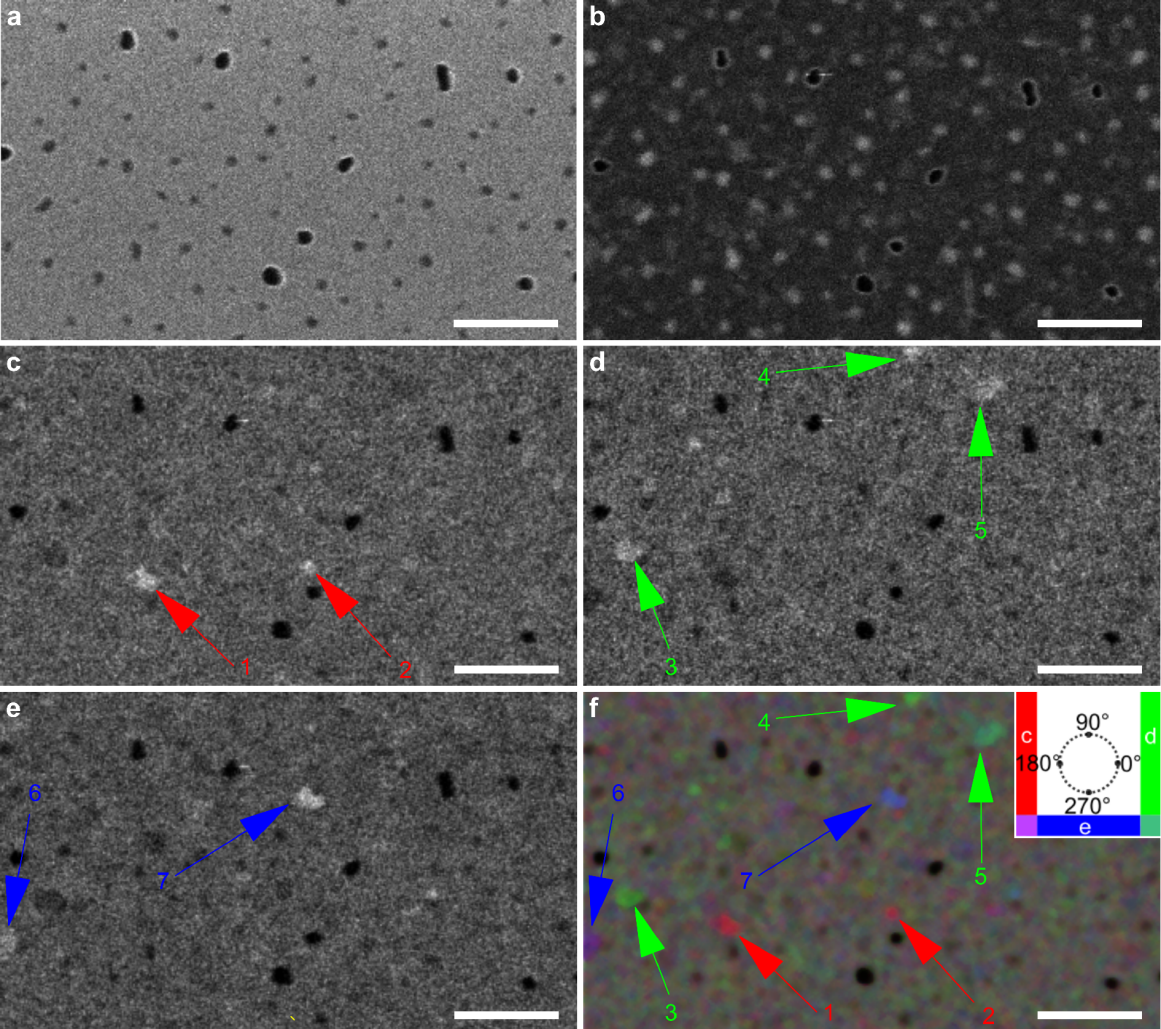
Helium ion microscopy images of the nanoporous polycrystalline silicon membrane. (a) SE image. (b) BF STIM image with polar angle θ *<* 3° and ϕ from 0 to 360°. Post-processed DF images with polar angle θ *>* 6° and azimuthal angle ϕ from (c) 135 to 225°, (d) 315 to 45°, (e) 225 to 315°. (f) Composite colored image using (c), (d), and (e) as RGB color channels. The inset shows the color mapping used in Figure 6f for the areas on the detector. The scale bars are 250 nm. The pixel dwell time used in the STIM data acquisition was 200 μs.

A 15 nm thick nanoporous polycrystalline silicon membrane (available from Electron Microscopy Sciences, item number: 76042–79) has been investigated using STIM. In Figure 4b–f we present several STIM images, which were generated by a single data acquisition process. Figure 4a corresponds to the same field of view in the SE imaging mode. In the SE image (Figure 4a), one can note that the bigger pores are completely black, since they are totally open and no signal comes from these areas. The smaller pores are possibly partially filled with carbon and yield some SE signal.

The bigger pores appear dark in dark-field mode (Figure 4c–e) because there is no scattering. Counterintuitively, the same pores appear dark in bright-field mode (Figure 4b) as well. This behavior can be explained by the intensity of the full primary beam exceeding the local rate capability of the detector. At a distance of 151 mm behind the sample, the beam diameter has only widened to 53 μm and quickly saturates the MCP pores with diameters of 25 μm, for a beam with 0.35 mrad convergence. The high local current density temporarily discharges the irradiated pores preventing the creation of further electron cascades above the discriminator value, resulting in dark pixels. Grains that are thinner than the others, and smaller pores partially filled with residual carbon, appear brighter than the average in BF and darker in DF as expected. This contrast is due to the reduced scattering that the ions undergo when passing through such an area. Figure 4c–e shows dark-field images created using the same polar angle but different azimuthal directions on the detector (different from annular dark-field images where all azimuthal angles are considered).

The regions indicated by the arrows show contrast variations in different azimuthal directions of detection, with the same polar angle. The size and shape of these regions are comparable to the size and shape of the grains of the sample. This contrast change can be explained by channeling and blocking effects. For a random orientation or an amorphous material the polar angle of the scattering would depend only on the mass-thickness product of the traversed material and no azimuthal pattern is expected. However, for crystalline materials, depending on the crystal orientation with respect to the beam, the ions can be channeled along a low-index crystal axis or plane and, as a result, are steered into a particular direction. In a STIM image composed of the intensity in this particular polar and azimuthal direction, the grain will appear brighter than the average (e.g., grain 3 in Figure 4d). Conversely, the same grain will show a lower intensity than a randomly oriented grain for other non-channeling directions, since the beam is not being scattered into random directions as much as it would be the case for a randomly oriented grains (grain 3 in Figure 4c and Figure 4d). Figure 4f is an RGB image created using the three different DF directions as color channels. Using appropriate azimuthal angles for the channels, this composite image shows the grains that are steering the beam to directions between two directions used for individual channels presented in Figure 4c–e. For instance, grain 5 appears as cyan (overlap between d and e) and grain 6 appears purple (overlap between c and e).

The exit-angle distribution depends on the channeling occurring in the crystalline grains the beam traverses. The best contrast for the grains is obtained at angles larger than the largest critical angle for silicon. Therefore, we can infer that the ions are not following the same channel from the beginning to the end. Since this effect would steer the beam to the angle between the crystal axis and the beam, having an upper limit equal to the maximum critical angle for channeling. In silicon, this value would be 3.51°, for the ⟨110⟩ directions, calculated using an adaptation of [40]. This is also the direction where the minimum backscattering yield (maximum transmission) is expected. Instead, the ions enter the crystal and, after some deviation due to random scattering, they reach directions in which they are channeled. Holeňák et al. [23] showed the blocking pattern of 50 keV helium through a 200 nm single-crystalline silicon foil at a pseudo-random orientation. In their report, some high-intensity spots were present at angles higher than twice the channeling critical angle.

#### Thallium chloride

A transmission electron microscopy (TEM) grid coated with evaporated thallium chloride (available on https://scienceservices.de/ with product code: Sku:E80045) was also analyzed using STIM. This sample has several small crystallites randomly oriented and it is used as a diffraction standard for TEM. Here, we perform a analysis similar to that carried out with the polycrystalline sample.

**Figure 5:**
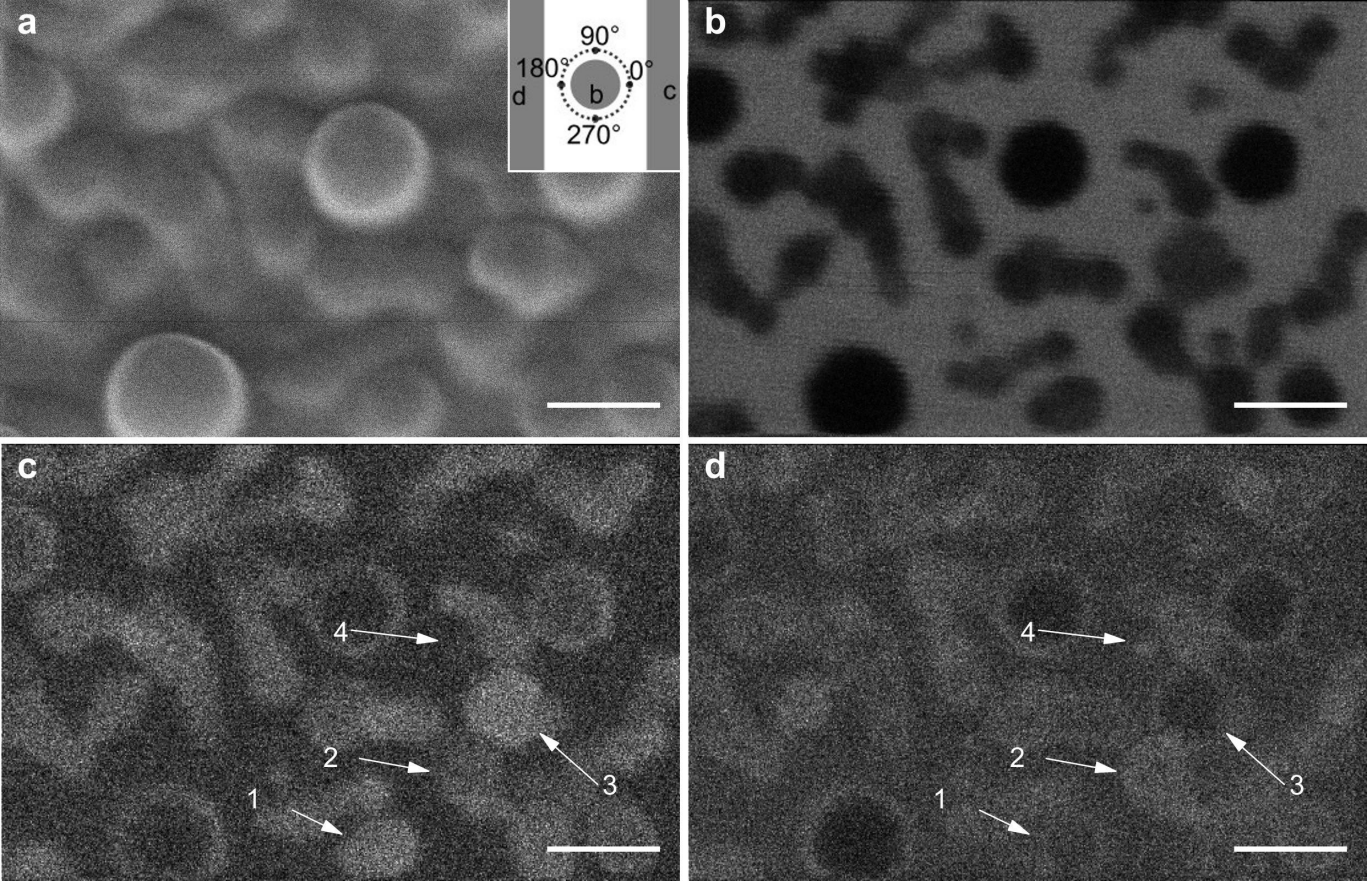
Thallium chloride evaporated on a TEM grid. (a) Secondary electron image. Inset of (a) shows the regions of the detector used to generate the following STIM images. (b) BF STIM image with acceptance angle of 0 to 4°. (c) DF STIM using the signal from the region on the right. (d) DF STIM using the signal from the region on the left. The scale bars are 100 nm.

The SE image presented in Figure 5a and the BF STIM image (Figure 5b) show crystallites with different sizes. Additionally, the BF image (Figure 5b) gives information on the size of the crystallite along the beam axis according to their intensity level. The DF images Figure 5c and Figure 5d were obtained using the same polar angle, but with opposite azimuthal directions.

A comparison between the size of the structures in the SE image (Figure 5a) and the BF image (Figure 5b) shows larger structures in the SE image. Considering that a thin film of a light material would show in the SE signal but would not increase significantly the scattering and, hence, would not appear in the BF STIM mode, we assume that there is a film of approximately 10 nm over the crystallites. As expected, most of the smaller crystallites that show as dark in BF are bright in DF. Some larger crystallites are dark both in bright-field and dark-field images because of their larger thickness, which causes the beam to be scattered to angles higher than the maximum angle covered by the detector. A fraction of the ions might be stopped since the thickness of some crystallites is comparable to the projected ion range of helium under the experimental conditions (estimated as 170 nm using SRIM [41]). There are, however, crystallites marked by arrows that show different intensity levels for different azimuthal directions. Crystallites 1 and 3 appear brighter in Figure 5c than in Figure 5d, while crystallites 2 and 4 behave in the opposite way. This difference would not occur in amorphous samples and can be explained with preferential scattering along low-index directions. Since the crystallites are randomly oriented, the axis in which the transmission of ions is enhanced points in different directions creating this variation of contrast for different azimuthal angles.

#### Single-crystalline silicon

**Figure 6:**
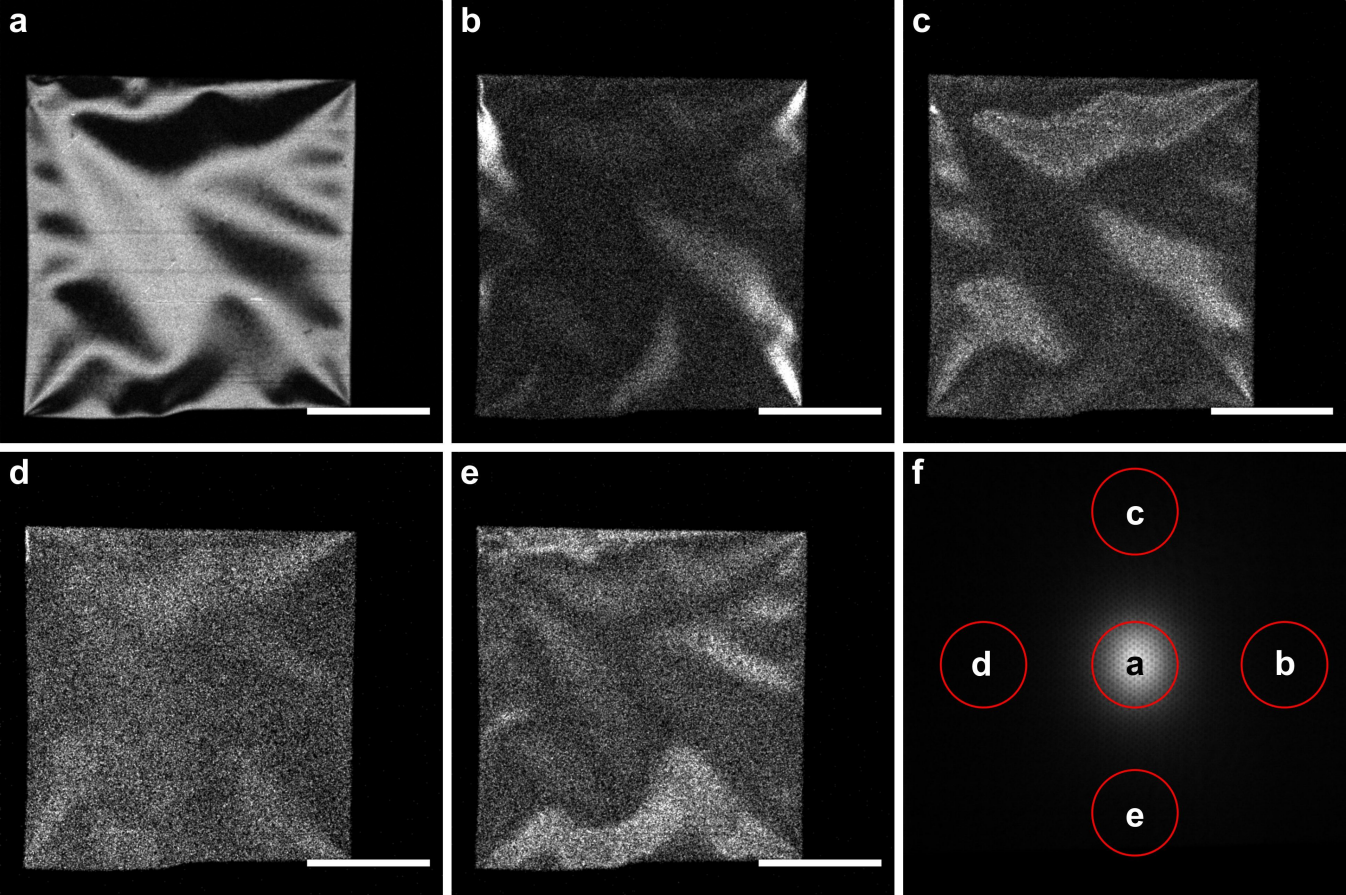
STIM images of a single-crystalline silicon ⟨100⟩ membrane in (a) bright-field with θ ≤ 1.09°, and in dark-field centered at the polar scattering angle θ = 3.8°, with azimuthal angle center ϕ = 0° in (b), ϕ = 90° in (c), ϕ = 180° in (d), and ϕ = 270° in (e). (f) Overall distribution of all counts on the detector. The areas on the detector for the corresponding STIM image are marked in red. The scale bars are 50 μm for (a–e). In (f), the distance from the center of the image to edge corresponds to a 5.58° deflection in the polar angle.

In Figure 6a–e, we show STIM using different sections of the detector and the image of the transmission signal (Figure 6). The sample was a 35 nm thick, ⟨100⟩-oriented silicon membrane window (available on http://TEMwindows.com, product code: US100-C35Q33). From Figure 6a, one can see that the membrane has wrinkles, which create different angles of incidence between the sample and the incoming beam. The images shown in Figure 6b–e are DF STIM images created using the same polar angles but different azimuthal angles. One can notice that the same areas of the sample show different contrast at different DF directions. This means that different areas of the sample scatter the beam in different preferential directions, depending on the local inclination of the film. Since the membrane is oriented in the ⟨100⟩ direction, we assume that channeling will predominantly occur along the same direction. The critical angle for 30 keV helium ions along this direction is 1.16°, calculated using an adaptation of [40]. Therefore, areas that are bright in dark-field images (Figure 6b–e) can be interpreted as areas in which this channeling direction points towards the corresponding dark-field region on the detector due to the local inclination of the film. If, for the given experiment, we assume that channeling will occur along the ⟨100⟩ direction close by, we can obtain the local tilt angle from the measured polar angles. The images presented in Figure 6 highlight the areas with a local tilt angle of 3.8°.

## Conclusion

In this work, we presented the development of a detection system for STIM that adds new functionalities to instruments based on the GFIS ion column, such as the helium ion microscope or other light ion beam methods with high lateral resolution. The system is based on the combination of MCPs and a delay line detector mounted on a movable support so that the experiment geometry can be optimized. The used imaging detector is capable of a random count rate of up to 5 Mcps and has a spatial resolution of approximately 50 μm. This detector has not shown performance degradation due to energetic particle damage even when exposed to the primary beam directly. Advantages of this detector over earlier approaches are its flexibility and numerous supported imaging modes. These include bright-field, annular dark-field, and dark-field for channeling applications. In the future the detector will also provide time-of-flight support for these modes with a temporal resolution of 200 ps (or 29 eV energy for our current setup using SE detection for the start signal). In addition, the concept provides the possibility for post-processing the recorded data into BF and DF according to the operator’s needs.

Using this detection system, we show applications of STIM for amorphous, polycrystalline, and single-crystalline materials. For amorphous samples, we show the contrast change for low and high scattering angles using BF and ADF detection. We also demonstrate the qualitative match of the contrast in bright-field mode with predictions from binary collision approximation calculations using a test sample. In the case of polycrystalline silicon, we can see channeling-related contrast in DF. Employing DF and post-processing, we see a contrast dependence on the orientation of thallium chloride nanocrystals. Finally, beam steering effects were shown to occur for a single-crystalline silicon sample.
